# Utilization of Research in Clinical Nursing and Midwifery Practice in Ghana: Protocol for a Mixed Methods Study

**DOI:** 10.2196/45067

**Published:** 2023-04-07

**Authors:** Lydia Boampong Owusu, Nicholin Scheepers, Immaculate Sabelile Tenza

**Affiliations:** 1 Department of Nursing, College of Health Sciences Kwame Nkrumah University of Science and Technology Kumasi Ghana; 2 School of Nursing Science North-West University Potchefstroom South Africa

**Keywords:** clinical practice, educators, evidence-based practice, health facilities, managers, midwife, midwives, nurses, nursing practice, nursing, research utilization

## Abstract

**Background:**

The International Council of Nurses’ 2021 code of ethics mandates nurses to provide evidence-informed care to patients. Globally, using research evidence has led to improvement in nursing and midwifery practice, according to the World Health Organization. A study in Ghana found that 25.3% (n=40) of nurses and midwives use research in clinical care. Research utilization (RU) increases therapeutic effectiveness, improves health outcomes, and enhances the personal and professional development of clinicians. However, it is uncertain the extent to which nurses and midwives are prepared, skilled, and supported to utilize research in clinical care in Ghana.

**Objective:**

This study aims to develop a conceptual framework that can facilitate RU among clinical nurses and midwives in Ghanaian health facilities.

**Methods:**

This will be a cross-sectional study with a concurrent mixed methods approach. It will be conducted in 6 hospitals and 4 nursing educational institutions in Kumasi, Ghana. The study has 4 objectives which will be executed in 3 phases. Phase 1 follows a quantitative approach to describe the knowledge, attitudes, and practices of clinical nurses and midwives on the use of research in their practice. Using a web-based survey, 400 nurses and midwives working in 6 health facilities will be recruited. Data analysis will be conducted using SPSS, with statistical significance set at .05. Qualitative methodology, using focus group discussions with clinical nurses and midwives, will be conducted to identify the factors influencing their RU. In phase 2, focus group discussions will be used to examine and describe how nurse educators in 4 nursing and midwifery educational institutions prepare nurses and midwives for RU during their education. Views of nurse managers on the RU in Ghanaian health care facilities will be explored in the second section of this phase through one-on-one interviews. Inductive thematic analysis will be used to analyze the qualitative data, and Lincoln and Guba’s principles of trustworthiness will be applied. In phase 3, the stages of model development proposed by Chinn and Kramer; and Walker and Avant will be used to triangulate findings from all objectives and formulate a conceptual framework.

**Results:**

Data collection started in December 2022. Publication of the results will begin in April 2023.

**Conclusions:**

RU in clinical practice has become an acceptable practice in nursing and midwifery. It is critical that nursing and midwifery professionals in sub-Saharan Africa shift their practice to embrace the global movement. This proposed conceptual framework will empower nurses and midwives to improve their practice of RU.

**International Registered Report Identifier (IRRID):**

DERR1-10.2196/45067

## Introduction

The 2021 code of ethics of the International Council of Nurses mandates that nurses across the globe should provide evidence-informed health care to patients [1]. This requires the use of research in practice. Consequently, according to the World Health Organization, using research evidence has led to improvement in nursing and midwifery practice globally [2]. Using research evidence is a component of evidence-based practice (EBP), which is a great means for nurses and midwives to meet the changing health needs of the society [3,4]. EBP is a systematic approach to problem-solving for health care providers [5] which is characterized by using the best research evidence that is currently available, clinical expertise, and patient values for clinical decision-making to provide consistent and best possible care to patients [6,7]. The transition to EBP not only increases therapeutic effectiveness and improves health outcomes but also enhances the personal and professional development of clinicians [4]. Research utilization (RU) is vital to the successful implementation of EBP [4].

RU is defined as the process of analyzing, disseminating, and implementing research-produced knowledge to influence or improve current nursing practice [8,9]. This can be achieved through nurses’ critical appraisal of research [10]. According to the World Health Organization, globally, nursing and midwifery services have been enhanced due to implementation of research evidence, which highlighted the significant role of nurses in patient care, especially for decision makers [2].

Globally, the use of research in nursing practice has been found to have enormous benefits for both providers and recipients of care [11]. The benefits extend to the public who receive care, the nurses and midwives who provide the care, and the health care system in which the health care is provided [12,13]. Nursing care that is based on the best available research evidence is vital to resolving problems in the clinical setting, improving patient outcomes, decreasing trial and error in nursing care, increasing nurses’ confidence in decision-making, and supporting professional development [14-17]. RU contributes to the science of nursing through the introduction of innovation to practice [15]. RU is critical in evidence-based nursing practice because it can save time and money by improving the quality of patient care through cost reduction and effective treatment [18]. It provides a more solid foundation for health care investment decisions and collaboration for capacity development [4]. In the nursing profession, research has been a vital means of developing new knowledge [2]. The benefits of RU in clinical nursing and midwifery practice cannot be realized if it is not implemented.

In Ghana, most of the studies that have been conducted on nurses and research have focused on EBP [14,19-21] with only 1 study [22] focusing on RU. The latter looked at the barriers to RU, given the scarcity of data on the knowledge, attitudes, and practices of RU by nurses and midwives in Ghana. There is methodological limitation in most of the studies on EBP and the RU study in Ghana because they used 1 methodology (either quantitative or qualitative). In addition, the findings of the studies on EBP cannot be used as RU because they are not the same and there are variations in their meanings. This has led to limited published studies in the literature on RU in Ghana.

In addition, there is an urgent need for formal training of nurses and midwives on how to utilize research evidence in their practice. However, there have not been any studies focusing on how nurses are prepared to implement RU in Ghana. This has led to a dearth of information on how well RU is incorporated in both the undergraduate and postgraduate curriculum and the extent to which they are prepared to implement RU after their training. Moreover, leadership plays a key role in the implementation of programs that ensure improved clinical care, which includes RU [23]. Nurse managers have been found to be influential on their staff in translating research evidence into action [24]. This creates a need to identify the views of nurse managers in Ghana on RU and how they can or have been influencing its practice in their health facilities.

Globally, research evidence use in nursing practice has become imperative due to increasing knowledge and technology [25]. Using research evidence in clinical care has not been fully achieved in nursing practice due to the inability of nurses to translate research into practice [26]. This has been confirmed by studies in Singapore [27], Brazil [28], Malaysia [29], South Africa [30], Ethiopia [31], Kenya [11] and Ghana [22]. This inability of nurses to translate research into practice has reflected on their use of research, as studies show Nigeria uses 17% of research [32]. In Kenya, 20.6% of nurses have participated in work-related research and half of them (10.3%) have ever implemented research evidence in practice [11]. In Ethiopia, poor RU was also found [33] and in Ghana, 25.3% research use was found [22]. This shows that if nurses are provided with guidance, research use will be improved.

Further, due to the enormous benefits of RU and the interplay of factors that influence its implementation, evidence suggests that the recurring problem of implementing research in clinical nursing practice can be resolved by offering a framework to guide the implementation of evidence in practice [11,18,22,34,35]. This study aims to establish such a framework to guide RU in clinical nursing and midwifery practice. This study seeks to explore RU by Ghanaian clinical nurses and midwives in hospital settings to develop a conceptual framework to guide RU in clinical nursing and midwifery practice. This will be achieved through the following objectives: (1) describe the knowledge, attitudes, and practices of clinical nurses and midwives toward the use of research in clinical practice in Ghana; (2) identify the factors (barriers or facilitators) that influence the use of research by nurses and midwives in clinical practice; (3) explore the preparation of nurses and midwives to implement RU in Ghana by nurse educators in nursing institutions; (4) explore the views of nurse managers on the implementation of RU in their facilities; and (5) develop a conceptual framework to guide nurses and midwives to utilize research in Ghanaian health facilities to improve the implementation of EBP in Ghana.

## Methods

### Study Design

The study will be cross-sectional, using a concurrent mixed method approach. The quantitative methodology will be used to identify the knowledge, attitudes, and practices of RU among the nurses. Concurrently, the researcher will collect qualitative data to explore the barriers to and facilitators of RU, the preparation of nurses and midwives, and the views of nurse managers on RU in health facilities. This will ensure synergy of the results to help in the development of a conceptual framework to facilitate RU in Ghanaian health facilities. This study will be implemented in 3 phases, chronologically. Quantitative data will be collected and analyzed independently, as well as the qualitative data. The results will then be interpreted and triangulated to inform the development of the conceptual framework of RU.

### Study Setting

The study will be conducted in health care facilities and nursing and midwifery educational institutions within Kumasi city, in the Ashanti region of Ghana. The study sites are 6 health facilities and 4 nursing educational institutions, depending on the study phase and objective. Specifically, the health facilities are 1 teaching hospital, 1 quasi-health facility, and 4 Ghana Health Service facilities. The facilities were selected for the study because each represents an agency of the Ministry of Health in Ghana, which regulates their activities. The educational institutions are 2 degree-awarding universities and 2 diploma-awarding training colleges. The institutions were selected based on ownership, that is, private or public, and the level of education—diploma or bachelor’s degree. This is to ascertain whether there are variations in the preparation of nurses and midwives based on the ownership and level of training.

### Phase 1

This phase will be divided into 2 sections based on the specific objectives to be achieved. The study sites for this phase are 6 health facilities.

#### Phase 1: Objective 1

##### Overview

The goal of the first part is to describe the knowledge, attitudes, and practices of clinical nurses and midwives regarding the use of research in clinical practice in Ghana. The quantitative research methodology will be used to achieve this objective.

##### Population and Sampling

Nurses and midwives who work in the clinical units of the health facilities with a minimum educational qualification of a diploma in nursing or midwifery and who have worked for not less than 2 years post qualification will be included in the study. Nurses and midwives who will be on leave at the time of data collection or are working on a part-time basis will be excluded. A sample size of 400 was calculated using Taro Yamane’s formula for calculating sample size and a 10% dropout rate. Multistage sampling, including proportionate and systematic sampling methods with a sampling interval of 9, will be used to achieve the target sample size in each health facility.

##### Data Collection

All the data for this PhD study will be collected by the primary investigator (LBO), who is also trained in qualitative and quantitative research methods. For this study phase, data will be collected using an adapted Evidence-Based Practice Questionnaire created by Upton and Upton [27], as permitted by the original authors. Knowledge or skills, attitudes, and practice are the 3 subscales of the tool, which are measured using a 5-point Likert scale, where 1=very poor, strongly disagree, or never, and 5=excellent, strongly agree, and always. In its original form, the overall Cronbach α is .87. Since this study focused only on RU, some questions were modified to focus on retrieving and implementing current research evidence in clinical practice. The modifications were based on RU literature and Rogers’ theory of diffusion of innovation, which guides the overall study. The Cronbach α for the modified tool is .84. Data collection will be done using a web-based survey after obtaining the consent of participants.

##### Quantitative Data Analysis

Quantitative data will be analyzed using SPSS (version 25.0; IBM Corp) and Stata (version 16; StataCorp). Exploratory data analysis will be carried out for the sociodemographic characteristics of the respondents. Inferential statistics will be used to ascertain the association between demographics and knowledge, attitudes, and practice levels. The chi-square test for independence and its alternative the Fisher exact test will be used to examine the association between categorical variables, while the nonparametric signed rank test will be used to examine the difference in the participants’ knowledge scores, and the Pearson and Spearman correlation coefficients will measure the degree of agreement between the scores. Structural equation model and factor analysis will be adopted to assess the effect of demographic characteristics (gender, educational level, age, and number of years of clinical practice) on RU in practice. Statistical significance will be established at *P* value of .05.

##### Validity and Reliability of Quantitative Data

To test the validity of the modified data collection tool, face validity was established by sending the questionnaire to 5 expert nurses and midwives who have a bachelor’s degree and work in the clinical area. Their inputs were used to modify the questionnaire. Pretesting was then carried out with 40 nurses and midwives, representing 10% of the sample size, who work in 2 health facilities located within the study area but not included in the study, using a web-based survey. Results of the pretest and inputs from the study supervisors as well as consultant statistician were used to modify the questionnaire. Cronbach α was calculated for the tool after modification to ensure reliability thereof. The overall Cronbach α factor for the tool was .8438, which is acceptable.

#### Phase 1: Objective 2

The second objective of phase 1 will be to identify barriers and facilitators influencing RU by nurses and midwives in clinical practice. Qualitative methodology, specifically focus group discussions (FGDs) that will be held via Zoom for each facility, will be used to achieve this objective. The population will be the same as those who participated in objective 1 with 6-8 participants per group as suggested by Busetto et al [36] and Ochieng et al [37]. A note will be placed on the survey to request respondents who are interested in participating in the FGD to add their phone numbers. Those who provide their phone numbers will be contacted and asked to sign the consent form. An interview guide with 2 sections, exploring barriers to and the facilitators of RU will be used to direct and facilitate the discussions. Each FGD will last between 60 and 90 minutes as recommended [37-39] and based on the exhaustion of questions on guide and responses from participants. Data collection and analysis will be done simultaneously, and collection will be stopped when data saturation is achieved.

### Phase 2

#### Overview

The study in this phase will be used to achieve objectives 3 and 4. It will take place in 2 settings with 2 populations as follows: nurse managers in the selected health facilities used in phase 1 and nurse educators in 4 nursing and midwifery educational institutions.

#### Phase 2: Objective 3

##### Overview

The objective here is to explore and describe nurse educators’ preparation of nurses and midwives toward RU in nursing and midwifery educational institutions. The phase 2 study sites will be the 4 nursing educational institutions stated earlier.

##### Sampling and Data Collection

The study will purposively sample nurse educators who have not less than 2 years of teaching experience and teach core nursing courses like pediatric nursing, medical and surgical nursing, neonatal nursing, public health nursing, and puerperium in the selected institutions. FGDs via Zoom will be held under the direction of an interview guide. The guide has 3 broad, open-ended questions to lead the discussions, with an emphasis on how the nurse educators prepare the nursing and midwifery students for RU, the challenges students experience in RU after nursing education, and the strategies that nurse educators could use to enhance RU in clinical practice. The number of participants per group, the duration of discussions, and the number of discussion sessions will be same as phase 1, objective 2.

#### Phase 2: Objective 4

##### Overview

The second part of phase 2 is aimed at exploring the views of nurse managers on the RU in health facilities using qualitative methodology.

##### Sampling and Data Collection

Nurse managers in the 6 health facilities used in phase 1 will be engaged in a face-to-face key informant interview through purposive sampling. The interviews will be guided by a semistructured interview guide to solicit the nurse managers’ views on RU in their various health facilities. Data collection and thematic analysis will be done simultaneously and terminated when data saturation is reached.

### Qualitative Data Analysis

Data will be thematically analyzed using Braun and Clarke’s thematic analysis process [28]. The recorded data will be transcribed. Codes will be generated in a systematic fashion across the entire data set and will be collated into themes and subthemes to generate a thematic “map” of the analysis.

### Trustworthiness

A total of 2 researchers will analyze the data independently and hold coding meetings until they reach intercoder agreement. Themes which emerge will be discussed with a group of participants to achieve member checking, thereby ensuring credibility. Also, method triangulation where the use of FGDs and individual interviews for data collection will be done so that the limitations of each method is counterbalanced whiles their strengths are projected [40]. Again, data from multiple sources like nurse managers, nurse educators, and clinical nurses and midwives will be used to enhance the credibility of the study [40,41]. Transferability will be achieved by providing a thick description of the research context and clear methodology to enhance potential for replication of the study. Verbatim transcriptions and use of quotations from collected data in reporting the study will ensure confirmability [40].

### Phase 3: Objective 5

This phase aims to develop a conceptual framework to facilitate RU by clinical nurses and midwives in Ghana. Findings from phases 1 and 2 will be triangulated and used to develop the conceptual framework. Data triangulation which uses data from multiple sources [41] will be performed in this study. This will be guided by Rogers’ theory of diffusion of innovation and the 4 stages of model development proposed by Chinn and Kramer (2008) and Walker and Avant (2005), ie, concept development, statement development, model description, and model evaluation.

### Ethical Approval

The study has been approved by the Health Research and Ethics Committee of North-West University in South Africa (NWU-00070-22-A1), the Ghana Health Service Ethics Review Committee (GHS-ERC 003/07/22), the Komfo Anokye Teaching Hospital Institutional Review Board in Kumasi (KATH IRB/AP/152/22), and the Committee on Human Research, Publication, and Ethics of the School of Medicine and Dentistry at the Kwame Nkrumah University of Science and Technology in Kumasi, Ghana (CHRPE/AP/762/22).

## Results

Data collection started in December 2022. It is envisaged that the results from phase 1 will unveil the knowledge, attitudes, and practices of clinical nurses and midwives regarding the use of research as well as factors influencing research use by nurses and midwives in clinical practice in Ghana. Phase 2 will identify the views of nurse managers on RU in nursing practice in their facilities and how nurse educators prepared student nurses and midwives to utilize research. Once all the findings from different groups of participants have been analyzed, we expect that the conceptual framework will be developed.

## Discussion

### Overview

Globally, RU is of utmost importance in health care and therefore cannot be downplayed [10-12]. A systematic review identified that most studies conducted on RU in nursing practice used the quantitative method and were conducted in Sweden, the United States, and the United Kingdom [42] with a dearth of literature on RU in Ghana. This study will unveil the activities of nurses and midwives in implementing research in their clinical practice. Also, the factors influencing clinical nurses’ and midwives’ RU will be unearthed. This will lead to minimizing the barriers and enhancing the facilitators to promote RU. Moreover, assessing the nurse managers’ views on RU will bring out how leadership in nursing influences RU in their various facilities. The study will identify how nurses are prepared for RU while they are in nursing school. This will guide the scope of the framework that will be developed. A systematic review of studies on barriers to RU in nursing practice from 2002 to 2021 by Jabonete and Roxas (2022) found that almost all studies on barriers to RU used the Barriers scale through a quantitative methodology. The review found that the Barriers scale is outmoded and therefore research on the barriers to RU requires a more robust approach to identify the challenges to nurses RU [42]. This study will use the qualitative methodology to identify the barriers to RU. The use of mixed methods will enable triangulation of findings, observation of the divergence and convergence of findings, and elimination of possible biases. This will also assist the researchers to examine the interplay of factors at the individual (clinical nurses and midwives), group (nurse educators), and organizational (nurse managers) levels [29]. Inclusion of nurse educators, managers, and clinical nurses and midwives as participants in the study contributes to the coproduction of knowledge and increases the chances of the acceptability and utilization of the framework. Nurses will use research if there is guidance on translating research into practice [31]. Consequently, several frameworks have been developed for RU [43]. However, few frameworks have been extensively used, in part because the dynamic processes and players involved are varied and context-specific but also because some frameworks offer little “how-to” guidance for implementation [44-46]. For an RU framework to be accepted for use, it must be contextually appropriate and suit the needs of countries with similar developmental classifications [45]. In the Ghanaian context, this is one of the first studies which focuses on RU. The conceptual framework that is developed will guide nurses and midwives on the implementation of RU in clinical care, guide the managers on how best they can support RU, and guide nurse educators on teaching of RU.

### Expected Clinical Impacts

The outcome of this study will have clinical, scientific, and socioeconomic impacts as it aims to produce a conceptual framework that will guide nursing and midwifery practice. The use of this framework will enhance the scientific practice of nursing and midwifery by enhancing evidence-based nursing care through the introduction of innovation to practice. Clinically, the outcome of this study, which is the development of the RU framework, when implemented, will improve patient outcomes by implementing tried and tested treatment modalities that will guide nurses and midwives’ decision-making and support their professional development. Socioeconomically, the implementation of the RU conceptual framework that will be developed at the end of this study will save time and money by improving the quality of patient care through cost reduction and effective treatment.

### Limitations

The study should be considered with the limitation that the study will not validate the conceptual framework that will be developed since it is beyond the scope of this study. However, we believe that the strong theoretical underpinning, the methodology, and the population used in the study, as well as the triangulation of the results of the study that will be used to develop the conceptual framework, will make the framework a must-use approach in clinical practice to enhance nurses and midwives RU. We again believe that future studies will be conducted to validate and pilot the use of the conceptual framework.

### Conclusions

RU in clinical practice has become an acceptable practice in nursing and midwifery. It is critical that nursing and midwifery professionals in sub-Saharan Africa shift their practice to embrace the global movement. This proposed conceptual framework will empower nurses and midwives to improve their practice of RU.

## Abbreviations

EBP: evidence-based practice
FGD: focus group discussion
RU: research utilization

## Data Availability

Data sharing not applicable to this paper as no data sets were generated or analyzed during this study.
